# Jiwa-style parenting and the familial turn of youth sport consumption: a qualitative case study of a commercial training setting in urban China

**DOI:** 10.3389/fspor.2026.1883178

**Published:** 2026-07-03

**Authors:** Yuanyuan Li

**Affiliations:** School of Physical Education, Sichuan Minzu College, Kangding, Sichuan, China

**Keywords:** community sport provision, family sport investment, jiwa-style parenting, participation equity, school physical education, youth sport consumption, youth sport training governance

## Abstract

**Background:**

Youth sport participation is increasingly shaped by family decisions, paid training opportunities, and unequal access to sport resources. Within urban Chinese family education contexts, jiwa-style parenting has extended beyond academic learning and entered children's sport participation, making training, equipment, competition experiences, and coaching part of family planning for child development. However, less is known about how this family logic operates within everyday commercial youth sport training settings.

**Methods:**

This qualitative case study draws on 20 days of participant observation and semi-structured interviews with 18 adult parents in one market-oriented youth fitness club in Guangzhou, China. The study used thematic analysis informed by grounded theory principles. Coding moved from repeated reading of Chinese interview transcripts and field notes, to initial coding, focused coding, theme development, and repeated comparison with the original materials. The analysis examined how parents interpreted the value of sport, how commercial training and school-related sport assessment shaped family decisions, and how differences in family resources affected access to sport opportunities.

**Results:**

Four interconnected themes were identified in this case. First, parents often reinterpreted children's sport participation as family sport investment linked to health, discipline, school-related assessment, and perceived future advantage. Second, some sport projects, equipment, and competition experiences became symbolic markers through which families expressed parenting competence and social distinction. Third, some parents became involved in pressure-driven training because of physical education assessment, peer comparison, and market promotion, even when they were concerned about cost, fatigue, or children's interest. Fourth, access to coaches, venues, training quality, and competition opportunities appeared to be differentiated by family resources.

**Conclusion:**

This study does not claim to represent youth sport consumption in urban China as a whole. Instead, it offers case-based qualitative insights into how youth sport consumption may be reorganized through family investment, symbolic distinction, pressure-driven training, and unequal opportunity access within one observed commercial training setting. The findings suggest that school physical education, community sport provision, and youth sport training governance should attend not only to participation expansion, but also to children's agency, family burden, and participation equity.

## 1 Introduction

Youth sport participation is often discussed as a matter of health promotion, physical literacy, character formation, social development, and lifelong engagement in physical activity. Yet participation is also shaped by the social and economic conditions that make sport opportunities available. Families decide whether children attend training, which activities they pursue, whether they compete, what equipment they use, and how much time and money can be devoted to sport. In this sense, youth sport participation is not only a pedagogical or public health issue; it is also a family resource allocation issue.

International research has increasingly shown that family culture, socioeconomic resources, and parental involvement matter for children's sport pathways (1–3). Organised sport can provide important developmental opportunities, but access to these opportunities is often uneven when participation depends on paid clubs, transport, equipment, coaching, and competition fees (4, 5). These studies suggest that youth sport should be examined not only as physical activity, but also as a social field in which family resources and aspirations are converted into participation opportunities.

In urban China, this family dimension has become particularly visible. Sport training, fitness courses, physical education examination preparation, competition registration, sport equipment, private coaching, and venue services increasingly enter family educational arrangements. Sport consumption is therefore moving beyond leisure and health consumption. It is becoming a form of family sport investment through which parents seek bodily development, discipline, confidence, teamwork, social skills, and potential future advantages for their children.

This process has also been shaped by recent changes in China's educational and sport context. The Double Reduction policy reduced the burden of homework and off-campus academic tutoring, while family demand for children's development did not disappear (6). Some family attention shifted toward non-academic activities, including sport, arts, and other forms of competence-building. At the same time, school physical education and health have gained greater policy visibility, and physical fitness tests, physical education examinations, after-school sport services, and youth sport competitions have become increasingly salient in family decision-making (7, 8). Commercial youth sport training institutions have expanded in this context by offering fitness courses, sport-skill training, examination-oriented preparation, competition pathways, and private coaching (9). These conditions make youth sport consumption an important site for examining how family aspirations, school-related assessment, and market provision intersect.

However, existing studies have not sufficiently explained how parents themselves interpret these sport-consumption decisions in everyday training settings. In particular, more qualitative evidence is needed on how parents connect sport training with all-round development, school assessment, peer comparison, symbolic display, and unequal access to coaching, venues, competitions, and sustained training support. This article addresses this gap through a qualitative case study of one commercial youth sport training setting in Guangzhou.

This article examines this process through the concept of jiwa-style parenting. The Chinese term jiwa is widely used to describe parenting practices marked by high expectations, heavy investment, close monitoring, and a concern that children should not fall behind. In this study, jiwa-style parenting is not treated as a simple label for anxious parents. Rather, it is understood as a family logic through which sport, academic learning, arts, and social abilities are incorporated into a planned and comparative portfolio of child development. When sport is drawn into this logic, it may be reinterpreted from everyday physical activity into strategically organised family investment.

Although jiwa-style parenting overlaps with international discussions of intensive parenting and concerted cultivation, it has a more specific meaning in the contemporary Chinese context. Intensive parenting emphasizes high parental involvement, intensive time investment, and the belief that parents are responsible for actively optimizing children's development (10). Concerted cultivation highlights how middle-class families use organized activities, institutional negotiation, and skill development to reproduce social advantage (11). Jiwa-style parenting shares these features, but it is more strongly marked by competitive comparison, anxiety about falling behind, and the incorporation of academic, artistic, physical, and social abilities into a planned portfolio of child development (12, 13). In this sense, jiwa-style parenting is not simply a psychological label for anxious parents. It refers to a family logic shaped by educational competition, peer comparison, and the pursuit of visible developmental advantage.

This distinction is important for the present study because sport is not traditionally understood only as academic preparation. When sport is drawn into jiwa-style parenting, it may be reinterpreted as a form of family educational investment. Training courses, equipment, competition experiences, certificates, and coaching may become linked to children's all-round development, school-related assessment, social display, and perceived future advantage. The analytical focus of this article is therefore not jiwa-style parenting in general, but how this parenting logic becomes expressed through youth sport consumption in one commercial training setting.

The familial turn of youth sport consumption is ambivalent. On the one hand, family attention to sport can increase participation opportunities and encourage parents to value physical fitness, motor skills, resilience, teamwork, and social development. On the other hand, when youth sport becomes closely tied to family competition, project hierarchies, examination preparation, competition results, and the ability to purchase better coaching or facilities, participation may become more utilitarian and unequal. Commercial training can offer additional opportunities, but it can also intensify family burdens and make access to high-quality sport experiences dependent on family capital.

Against this background, the present study examines how jiwa-style parenting is expressed within one commercial youth sport training setting in Guangzhou. The literature gap addressed by this study is twofold. First, although previous research has examined parental involvement, family resources, and socioeconomic disparities in youth sport participation, less is known about how sport is incorporated into Chinese families' broader educational planning under jiwa-style parenting. Second, existing discussions of commercial youth sport training often focus on market expansion, policy regulation, or participation outcomes, while paying less attention to parents' everyday meaning-making within training settings.

The originality of this study lies in connecting jiwa-style parenting, family sport consumption, symbolic distinction, and unequal access to sport opportunities within one qualitative case. By doing so, the study contributes case-based knowledge about how family expectations, school-related sport assessment, peer comparison, and commercial training provision may jointly reshape youth sport participation.

**Figure 1:**
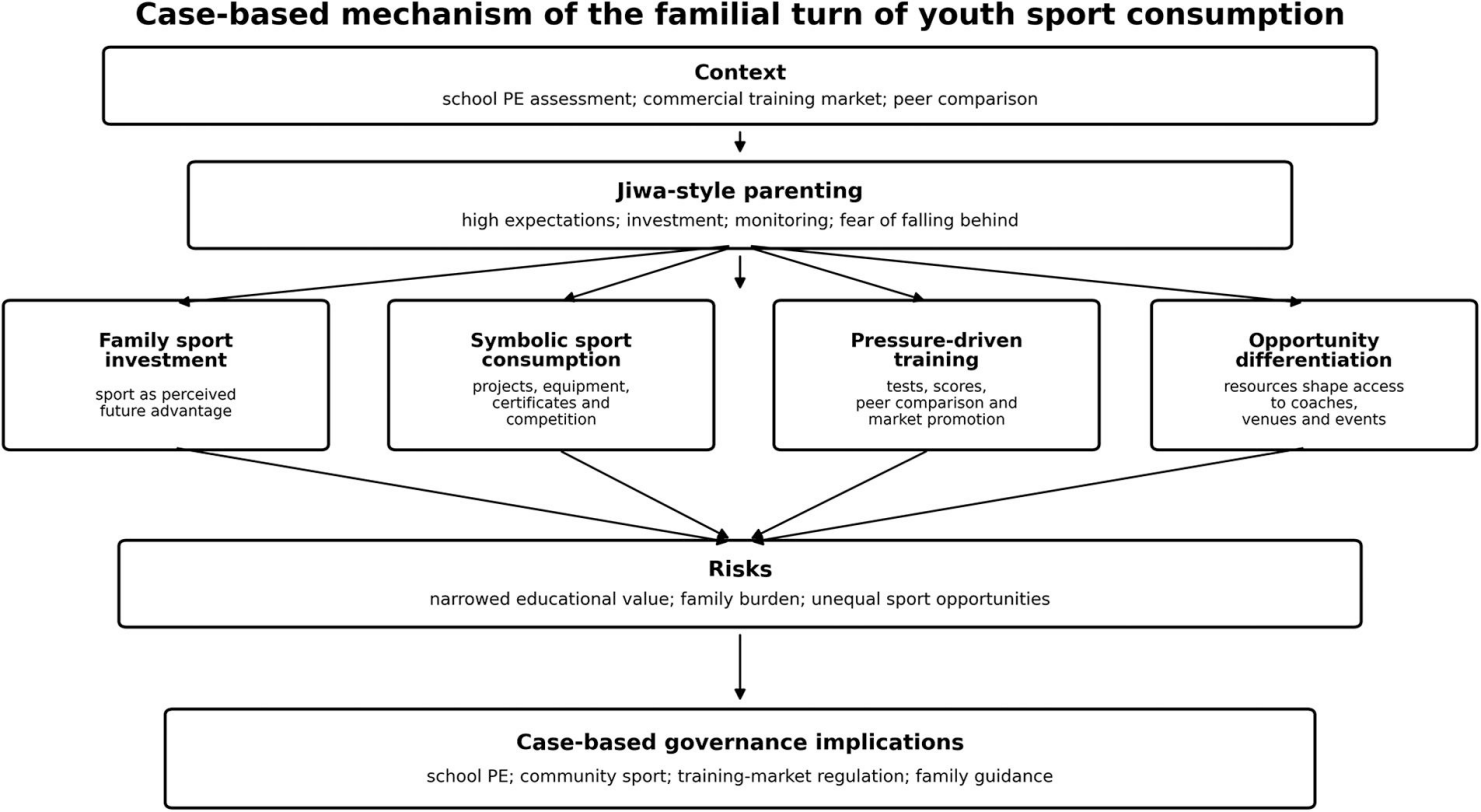
Case-based mechanism of the familial turn of youth sport consumption.

The study is guided by three research questions. First, how did parents in the observed commercial youth sport training setting interpret the value of sport participation for their children? Second, how did family expectations, peer comparison, school-related sport assessment, and commercial training practices shape parents' sport-consumption decisions? Third, what case-based implications can be drawn for school physical education, community sport provision, and youth sport training governance? By addressing these questions, the study contributes qualitative evidence from one urban Chinese training context and develops a mechanism-oriented interpretation of the familial turn of youth sport consumption. The case-based mechanism developed from this analytical framework is summarized in Figure 1.

## 2 Literature review and analytical framework

### 2.1 Family support and youth sport participation

Youth sport participation is not determined only by children's interest or school provision. Parents provide transport, pay fees, purchase equipment, encourage participation, select clubs, communicate with coaches, and decide whether children continue or withdraw. Family support can therefore enable sustained participation, especially when organised sport requires time, money, information, and emotional labour (1, 2).

However, family support is not always neutral or developmentally beneficial. Its meaning depends on the expectations and decision-making logic behind it. Research on parental involvement in youth sport has shown that parents may offer emotional, logistical, and financial support, but parental expectations and pressure can also shape young athletes’ experiences (1, 14). Studies based on young people's own accounts further suggest that adolescents often distinguish supportive involvement from intrusive involvement, especially when parental involvement constrains autonomy and peer sociability (15). A sport-governance perspective therefore needs to examine not only whether families support youth sport, but how such support is organised and what consequences it has for participation equity and young people's experiences.

### 2.2 Youth sport consumption and family sport investment

Sport consumption includes training services, venues, equipment, competitions, sport tourism, digital services, and health-related products. For children and adolescents, sport consumption is usually mediated by parents. It is the family that purchases training packages, arranges schedules, chooses clubs, pays for competitions, and decides whether a project is worth sustained investment. Compared with adult sport consumption, youth sport consumption therefore has stronger family decision-making and developmental-investment characteristics.

Under jiwa-style parenting, sport consumption is often interpreted as investment in children's future development. Sport ability may represent physical competence, self-discipline, teamwork, confidence, social skills, and sometimes a resource that can be displayed in school or family networks. This interpretation makes parents willing to invest money, time, and emotional labour in organised sport training. The key analytical issue is not simply the expansion of a consumer market, but how sport becomes embedded in family strategies of child development. This focus also distinguishes the present analysis from a purely educational-policy account: the empirical object here is the changing organisation of youth sport participation through family consumption.

### 2.3 Family capital, sport opportunity, and social distinction

Bourdieu's concepts of capital, habitus, and distinction provide a useful lens for understanding youth sport consumption (16, 17). Economic capital shapes whether families can afford high-quality coaching, premium venues, specialist equipment, and competition opportunities. Cultural capital shapes parents' understanding of sport's educational value, the relative prestige of sport projects, and the bodily dispositions they hope children will acquire. Social capital affects access to information about clubs, coaches, competitions, and peer networks. Symbolic capital can be accumulated through certificates, competition experiences, and participation in socially valued sport projects.

International evidence shows that family resources are associated with organised sport participation, club sport access, and specialised sport pathways. For example, research on social class differences in youth sport participation highlights the mechanisms through which socioeconomic resources shape access to organised sport (4). Reviews of participation equity also show socioeconomic disparities in organised sport and physical activity among children and adolescents (5). Studies of club sport and sport specialisation further indicate that higher parental income and education are linked to access to more intensive and specialised sport experiences (18, 19).

From this perspective, youth sport consumption is not merely a question of which sport a child learns. It is also a process through which families display parenting competence, educational planning, and social position. When some projects are described as high-end, distinctive, or worth showing, sport consumption can become a vehicle of social distinction. Differences in family capital may then translate into differences in sport opportunities, making youth sport participation a field where public educational ideals and private family resources intersect. Similar concerns about family educational investment, socioeconomic inequities in youth physical activity and sport, and class differentiation in urban family physical education have also been discussed in related studies (22–24).

## 3 Materials and methods

### 3.1 Research design

This article reports an exploratory qualitative case study based on participant observation, semi-structured interviews, and field notes. A qualitative case-study design was appropriate because the study aimed to understand parents' meaning-making, family decision-making, and the mechanisms through which youth sport participation was reorganized as a form of family-mediated consumption. The purpose was not to estimate the prevalence of these patterns in urban China or to generalize statistically to all youth sport training settings. Rather, the study uses one theoretically informative case to develop a mechanism-oriented interpretation of how family expectations, commercial youth sport training, and unequal access to sport resources may interact in an everyday urban training context.

The study was informed by thematic analysis and grounded theory principles (20, 21). Thematic analysis was used to identify patterned meanings across interviews and field notes, while grounded theory-inspired coding supported an iterative movement between empirical materials and conceptual interpretation. The analysis was also sensitized by literature on intensive parenting, family capital, social distinction, and youth sport participation, but the final themes were developed primarily through engagement with the empirical materials.

### 3.2 Field site

The field site was a market-oriented youth fitness club in Guangzhou, a major city in southern China. The club provided paid training services for children and adolescents, including physical fitness training, basketball, skipping, basic swimming-related training, competition preparation, and general sport ability development. It operated outside the free school-based after-school service system and relied mainly on family payment for training packages, course renewal, equipment, and competition-related services.

During the observation period, the club served children from preschool age to adolescence. Training was usually organized through small-group sessions, while some parents also discussed more intensive or individualized coaching. Families' expenditure included ordinary course packages, course renewal, sport equipment, competition registration, and, in some cases, more individualized forms of training support. Because the study was not designed as an institutional audit of the club, exact enrolment, revenue, and complete fee records were not collected. The club should therefore be understood as an ordinary market-oriented commercial training setting observed for qualitative analysis, rather than as a statistically representative institution or an elite sport academy.

This site was selected because it provided a suitable setting for observing how parents discussed, purchased, evaluated, and compared youth sport training. Parents commonly stayed in waiting areas, communicated with coaches, observed children's training, discussed school physical education tests, compared sport projects, and exchanged information about fees, coaches, competitions, and children's progress. The club should not be treated as representative of all youth sport training institutions in Guangzhou or China. Rather, it is a theoretically useful case because it made visible the interaction between family expectations, commercial training provision, school-related sport assessment, and parental comparison. The findings are therefore interpreted as case-based qualitative insights rather than general evidence about all urban Chinese families.

### 3.3 Participants and sampling

The study used purposive and snowball sampling. After permission was obtained from the club, the researcher conducted open participant observation and gradually built trust with parents in the training setting. Parents were invited to participate if they met three criteria: they were adult caregivers of children or adolescents involved in sport training; they had experience of making sport-consumption decisions for their children; and they were willing to discuss their parenting beliefs, training choices, perceived pressures, and expenditure-related experiences.

Eighteen adult parents participated in semi-structured interviews. Participant characteristics are presented in Table 1. They varied in gender, age, occupation, number of children, and children's ages. The number of parents who declined participation was not systematically recorded, because recruitment occurred gradually through field contact and informal introduction rather than through a fixed sampling frame. This is acknowledged as a limitation. The sample was sufficient for the purpose of this exploratory qualitative case study because the interviews and observations generated repeated patterns around sport as family investment, symbolic sport consumption, pressure-driven training, and differentiated access to sport opportunities. During the later interviews, few substantially new themes emerged, suggesting that thematic saturation had been approached within the scope of this case.

**Table 1:** Participant characteristics.

ID	Parent gender	Parent age	Occupation	Number of children	Child age(s)
P01	Male	35	Doctor	2	8, 10
P02	Female	44	University teacher	1	20^a^
P03	Female	31	Fitness coach	1	8
P04	Male	32	Photographer	1	5
P05	Male	40	Secondary school teacher	1	17
P06	Female	38	Homemaker	1	14
P07	Male	42	Bank employee	2	14, 16
P08	Female	34	Newspaper editor	1	7
P09	Female	39	Psychological counsellor	1	11
P10	Male	39	Programmer	2	8, 10
P11	Male	27	Company employee	1	4
P12	Male	43	Civil servant	1	9
P13	Female	45	Accountant	1	18^a^
P14	Female	35	Company employee	1	7
P15	Female	38	Business owner	1	11
P16	Female	46	Accountant	1	15
P17	Female	37	Secondary school teacher	1	13
P18	Female	30	Company employee	1	7

a Indicate parents’ retrospective accounts of sport training during basic education.

Two participants provided retrospective accounts involving children aged 18 and 20. These accounts were not used to make direct comparisons with parents of younger children currently enrolled in training. Instead, they were used cautiously as contextual materials to understand how parents reflected on sport training decisions during children's basic education. In the Results section, these retrospective accounts are treated as supplementary evidence and are not presented as equivalent to current observations of younger children's training.

The study did not systematically collect household income, parental education level, or exact family sport expenditure from all participants. Therefore, family capital was not measured as a statistical variable. Instead, it was inferred qualitatively from participants' occupations, narratives about affordability and training choices, observed consumption practices, project selection, training frequency, discussions of fees, and access to coaching, venues, equipment, and competition opportunities. Accordingly, the analysis of participation equity should be understood as interpretive and exploratory rather than as a direct measurement of socioeconomic inequality.

### 3.4 Data collection

Data were collected through 20 days of participant observation, semi-structured interviews, and field notes. Observation focused on training sessions, parent waiting areas, parent-coach interactions, course renewal conversations, project selection, and parents' informal discussions about school physical education, examinations, competitions, training costs, equipment, and children's responses to training. Field notes were written after each observation session and included descriptions of observed interactions, informal conversations, emerging analytical ideas, and the researcher's reflections.

Semi-structured interviews lasted approximately 30–50 min. The interview guide covered five main areas: why parents chose sport training for their children; how sport was positioned in children's development plans; how school physical education tests, entrance examinations, or competitions influenced family decisions; what pressures or burdens sport consumption created; and how parents perceived children's interest, fatigue, enjoyment, or resistance. No minors were directly interviewed. Any claims about children's experiences are therefore based on parental reports and observational impressions, rather than children's own interview accounts.

The interviews were conducted in Chinese. Interview notes and transcripts were first reviewed and coded in Chinese to preserve the original meanings of participants' expressions. Quotations selected for the English manuscript were translated by the author. During translation, the author compared the English versions with the Chinese originals to ensure semantic accuracy, especially for culturally specific expressions such as jiwa, interest classes, all-round development, face, and not falling behind. Where literal translation could create misunderstanding, the meaning was rendered in idiomatic academic English while preserving the participant's intended meaning.

### 3.5 Data analysis

Data analysis followed an iterative coding process. First, the researcher repeatedly read the interview transcripts and field notes to become familiar with the materials. Second, initial codes were generated close to the data. These included, for example, all-round development, physical education examination, training return, peer comparison, parent-circle pressure, project status, branded equipment, course renewal, financial pressure, being unable not to enroll, and concern about children's fatigue. Third, related initial codes were grouped through focused coding. For example, all-round development, training return, and perceived future advantage were grouped under family sport investment; peer comparison, project status, and parenting taste were grouped under symbolic sport consumption.

Fourth, focused codes were further organized into broader axial themes. Four main themes were developed: family sport investment, symbolic sport consumption, pressure-driven sport training, and differentiation of sport opportunities. Fifth, the themes were checked against the original interview and observation materials through constant comparison. The researcher compared cases that strongly supported a theme with cases that complicated or qualified the theme. This process helped avoid treating all parental involvement as pressure or all sport consumption as inequality. Finally, representative quotations and observational examples were selected to show the movement from empirical materials to interpretation. Examples of this coding movement are presented in Table 2.

**Table 2:** Examples of coding movement from empirical materials to initial codes, axial themes, and interpretive meanings.

Data source	Empirical material example	Initial code	Axial theme	Interpretive meaning
Interview	“I pay more attention to my child's all-round development.”	all-round development; ability reserve	Family sport investment	Sport was interpreted as part of a broader family plan for child development.
Interview	“When it comes to the PE examination, we must try to get full marks.”	examination preparation; training return	Family sport investment	Sport training was linked to school-related assessment and perceived future advantage.
Observation	Parents asked whether children were suitable for advanced courses or competition opportunities.	course upgrading; certificates; competition pathway	Family sport investment	Training progress was monitored and evaluated as a family investment chain.
Interview	“My neighbor's child goes to four interest classes every week..”	peer comparison; parent-circle pressure	Symbolic sport consumption	Sport choices were shaped by parent networks and comparison with other families.
Interview	“If the child has no interest class or special skill, there is no way to compare.”	special-skill anxiety; parenting taste	Symbolic sport consumption	Sport projects became markers of parenting competence and social distinction.
Field notes	Parents discussed fencing, skiing, dance, equestrian activities, and specialized swimming as more distinctive or presentable than basic activities.	project hierarchy; presentable projects	Symbolic sport consumption	Some projects acquired symbolic value beyond children's immediate interest or health needs.
Interview	“If we do not hurry to enroll in extra training, the score will be affected.”	unable not to enroll; examination pressure	Pressure-driven training	Parents described training as a risk-management response to assessment pressure.
Interview/observation	Parents reported high fees, mechanical training, and children's fatigue, while still considering continued enrolment.	ambivalence; fatigue; market pressure	Pressure-driven training	Participation was shaped by tension between concern for children and fear of falling behind.
Interview	“Skipping-rope training can improve scores, but I cannot afford it.”	fee pressure; limited resources	Opportunity differentiation	Family resources shaped whether parents could purchase additional training support.
Observation/field notes	Families discussed course packages, private coaching, equipment, competition registration, and venue quality.	cost items; information access; training quality	Opportunity differentiation	Access to sport opportunities differed by economic, cultural, and informational resources.

The coding process was primarily inductive because the themes emerged from repeated engagement with the empirical materials. At the same time, the analysis was theoretically sensitized by concepts such as intensive parenting, family capital, and social distinction. These concepts did not predetermine the coding categories, but they helped interpret why sport training became meaningful as family investment, why some projects acquired symbolic value, and why unequal resources shaped access to sport opportunities.

### 3.6 Trustworthiness, reflexivity, and researcher positionality

Several strategies were used to strengthen trustworthiness. First, data triangulation was conducted across interviews, participant observation, and field notes. Second, constant comparison was used to examine similarities and differences across participants, rather than relying on isolated or unusually strong statements. Third, typical quotations and observational details were used to connect conceptual interpretation with empirical materials. Fourth, negative or qualifying cases were considered, including parents who valued free play, questioned commercial training, or felt ambivalent about intensive sport participation.

Researcher reflexivity was also important because the researcher's presence in the club and interactions with parents may have influenced what participants chose to share. The researcher entered the field as an academic observer interested in youth sport participation and family sport consumption, rather than as a coach, club manager, or service provider. During observation, the researcher avoided intervening in training decisions, course sales, or parent-coach negotiations. At the same time, the researcher's academic background in school physical education and youth sport governance may have made issues of educational value, family burden, and participation equity especially salient during interpretation. Reflexive notes were therefore kept to record how the researcher's assumptions about school physical education, family investment, and youth sport governance might shape interpretation. These notes were used during analysis to distinguish between participants' own accounts, observed practices, and the researcher's conceptual interpretation.

### 3.7 Ethics and consent

The study involved adult human participants who discussed their own parenting beliefs and sport-consumption decisions. No minors were directly interviewed, and no identifiable images or personal data of children were collected for publication. Before each interview, participants were informed of the research purpose, the voluntary nature of participation, the intended academic use of the data, confidentiality measures, and their right to withdraw. Participants provided verbal informed consent before each interview.

The study was considered low risk because it involved interviews with adult participants and observation of a public-facing commercial training setting without intervention in children's training or collection of sensitive identifiable data. According to the institutional practice of Sichuan Minzu College for low-risk social science research involving adult participants, formal ethics review was not required for this study. All participant names were replaced with codes from P01 to P18. Identifying information about children, schools, the club, coaches, and specific events was removed or blurred in reporting.

## 4 Results: the familial turn of youth sport consumption

### 4.1 Sport participation as family investment: from health exercise to perceived future advantage

In the parent waiting area, parents often discussed physical education entrance examinations, fitness-test performance, training progress, course upgrades, and competition opportunities. Some parents asked coaches whether their children were suitable for advanced courses, whether additional training would improve test performance, and whether competition participation or certificates could become useful evidence of children's development. Such interactions showed that sport training had entered a family investment chain that was planned, monitored, and evaluated.

Many parents did not understand youth sport training simply as leisure or interest. They placed it within a broader plan for child development. P07 said, “I pay more attention to my child's all-round development. In today's society, only all-round talents can stand better.” P16 explained that because the child was learning basketball, “when it comes to the physical education examination, we must try to get full marks.” P05 also emphasized that sport activities could help children develop personality and, more importantly, teamwork and social competence. These accounts suggest that parents interpreted sport ability as part of children's overall competence, rather than only as physical exercise.

Sport training was therefore not merely an embodied experience in the present. It was incorporated into a perceived now-investment/future-advantage logic. Parents paid training fees, purchased equipment, arranged time, and monitored progress because they hoped sport would generate visible or potential returns. These returns included fitness-test scores, physical education examination preparation, certificates, competition experience, and materials for broader evaluations, as well as less quantifiable benefits such as perseverance, confidence, teamwork, and social skills. To avoid overstating the evidence, this study treats “future advantage” as parents' perceived expectation rather than as an objectively verified outcome.

Jiwa-style parenting did not marginalize sport. Instead, in this case, it reinterpreted sport through higher expectations and stronger planning. Sport was given multiple meanings: bodily development, character formation, school-related preparation, and perceived developmental advantage. This conversion into family investment created additional participation opportunities for some young people, but it also made sport training more likely to be organized around outcomes and external returns.

### 4.2 Symbolic sport consumption: from interest projects to family distinction

The familial turn also appeared in the symbolic reconstruction of sport projects, equipment, and competition experiences. During observation, parents often evaluated sport projects in hierarchical terms. Compared with basic activities such as skipping or running, projects such as fencing, skiing, dance, equestrian activities, and specialized swimming were more likely to be described in parent conversations as distinctive, presentable, or more suitable for display in school or family networks. These descriptions did not mean that such sports were inherently superior. Rather, they showed that some parents attached symbolic value to particular projects because they appeared to signal family resources, parenting taste, or a more refined form of child development.

Observational materials further suggested that symbolic sport consumption was not limited to project choice. Parents also discussed branded equipment, competition participation, certificates, and whether a child had a “special skill” that could be mentioned in school or parent-circle conversations. In these discussions, sport became linked to visible evidence of family investment. Competition results and certificates were sometimes treated not only as feedback on sporting performance, but also as signs that parental investment had produced a presentable outcome.

Peer comparison was an important driver of symbolic sport consumption. P07 said, “My neighbor's child goes to four interest classes every week, while ours only goes to two.” P18 noted that the family initially wanted the child to grow happily, but later felt that if the child's academic performance was ordinary and the child had no interest class or special skill, there would be no way to compare with other children. Such statements indicate that sport-consumption decisions were not based only on children's interest or physical development needs. They were also shaped by parent networks, neighborhood comparison, and social evaluation.

The symbolic meaning of sport consumption was also reflected in competition results and certificates. P13 had invested considerable money and time in the child's swimming training but felt embarrassed when competition results were not ideal. In this account, competition results were not simply feedback on a child's sporting performance; they also became a measure of whether parental investment had produced a presentable outcome. Sport participation was embedded in family face, parenting competence, and social comparison.

These findings suggest that, within this case, sport participation could become embedded in family face, parenting competence, and social comparison. Youth sport consumption was therefore not only about which sport children learned; it was also about how some families displayed positions and parenting effort through sport projects, equipment, and competition experiences. When sport projects acquire the function of social distinction, sport consumption may move away from children's enjoyment and health development toward symbolic competition among parents.

### 4.3 Pressure-driven involvement in sport training: from autonomous participation to risk management

Not all parents actively endorsed intensive sport training. Observation and interviews showed that some families complained about high fees and mechanical training. Some parents also reported that their children felt tired, showed reduced interest, or resisted repetitive training. Because no children were directly interviewed, these findings should be understood as parental reports and observational impressions rather than direct evidence of children's internal experiences. This distinction is important for interpreting the relationship between parental decisions and children's agency.

Fitness tests and physical education examinations were key sources of pressure. P06 said, “Now the child faces the physical education entrance examination. If we do not hurry to enroll in extra training, the score will be affected.” P14 added that examination scores could not simply be bought with money. These accounts show that sport training was incorporated into examination preparation. Even when parents were concerned about cost or children's willingness, some still considered continued training because sport scores were understood as resources that could not be lost.

Some parents were reflective about commercial sport training. P06 admitted that skipping-rope training could improve scores but that the cost was unaffordable. P09 preferred children to play freely and exercise in natural environments rather than receive mechanical training in commercial classes. Parents, then, did not necessarily accept the logic of intensive training. However, under the combined influence of fitness tests, entrance examinations, peer comparison, and market promotion, they could still be drawn into continued consumption.

This finding suggests that the expansion of youth sport training in this case cannot be explained only by parental demand or market supply. It must also be understood in relation to sport evaluation, schooling imagination, and family risk management. When sport participation is transformed into a task of not losing points, commercial training can shift from being a supplementary participation channel to a field that creates training pressure and family expenditure burden.

### 4.4 Differentiation of sport opportunities: family capital and participation equity

The familial turn of youth sport consumption also produced differentiation in sport opportunities. In the observed setting, high-quality coaching, specialist courses, course renewal, private coaching, competition registration, equipment, and premium venues all involved recurring costs. Although this study did not systematically collect exact household income or complete fee records, parents' narratives and field observations showed that these cost items shaped how families evaluated and sustained children's sport participation.

Some parents noted that high-level coaching and quality venues were expensive and difficult for middle- and lower-income families to sustain. When children had weaker physical foundations and also faced sport assessment pressure, families became especially anxious: not enrolling might affect scores, while enrolling created financial strain. P06's statement that skipping-rope training could improve scores but was unaffordable reflects the structural dilemma of resource-limited families in youth sport consumption.

Opportunity differentiation was not limited to whether children could attend training. It also concerned training quality, project choice, information access, and competition opportunities. Parents with greater family resources appeared more able to obtain information about training institutions, select prestigious projects, purchase equipment, renew courses, and support competition participation. Parents with fewer resources could be pushed toward cheaper, shorter, or more test-oriented training services. In this way, youth sport participation, which should retain public and inclusive educational value, may be re-stratified through marketized training.

The familial turn of youth sport consumption is therefore not only a market expansion issue. In this case, it also appeared as a participation-equity issue. If school physical education and community sport provision are insufficient, dependence on commercial training may deepen, and family-resource differences may be converted into unequal sport development opportunities. This interpretation should be read as qualitative case evidence rather than as a direct measurement of socioeconomic inequality.

## 5 Discussion

### 5.1 Familialization as a case-based mechanism

The main new finding of this study is that, within the observed commercial youth sport training setting, youth sport consumption was not organized only as leisure, health promotion, or skill learning. It was also reorganized through a family logic of educational investment, symbolic distinction, risk management, and unequal opportunity access. This qualitative case study therefore suggests a possible mechanism through which youth sport participation may become familialized within a commercial training context.

The originality of this study lies in showing how jiwa-style parenting can extend into youth sport and reshape parents' interpretation of sport participation. In relation to the first research question, parents interpreted sport as a way to support health, discipline, confidence, school-related assessment, and perceived future advantage. In relation to the second research question, peer comparison, project hierarchy, assessment pressure, and commercial training practices helped explain why some parents became involved in intensive or ambivalent forms of sport consumption. In relation to the third research question, the findings suggest case-based implications for the responsibilities of schools, communities, families, and commercial training providers.

The significance of the findings is that they link youth sport consumption with family education beliefs and equitable access to sport opportunities. Rather than treating commercial sport training only as a market service or an individual family choice, the study shows how sport opportunities may be shaped by the interaction of family resources, school-related assessment, peer comparison, and market provision. This does not mean that all urban Chinese families understand youth sport in the same way; rather, it identifies a mechanism that became visible in one observed commercial training setting.

### 5.2 Parental support, parental pressure, and market pressure

The findings also suggest the need to distinguish parental support, parental pressure, and market pressure. Parental support refers to the emotional, logistical, and financial assistance through which families enable children's sport participation. In this study, many parents valued sport because it could improve health, discipline, confidence, teamwork, and social competence. Such support can expand children's opportunities to participate in sport and reflects parents' growing recognition of sport's educational value.

Parental pressure refers to situations in which support becomes strongly tied to comparison, performance, assessment results, certificates, or the fear that children may fall behind. In the observed case, some parents did not simply encourage participation; they monitored progress, compared projects, worried about physical education examinations, and evaluated whether sport training produced visible returns. This pressure did not always come from parents' personal preference alone. It was often connected to school-related assessment, peer comparison, and broader family anxiety about children's development.

Market pressure refers to the role of commercial training institutions, course packages, coaching services, competition pathways, equipment consumption, and promotional claims in shaping family decisions. Commercial training can provide additional opportunities, especially when school and community sport provision is insufficient. However, when market services are framed around score improvement, course upgrading, competition experience, and visible outcomes, they may intensify family expenditure and reinforce the idea that better sport opportunities must be purchased. The interaction among parental support, parental pressure, and market pressure helps explain why some families became involved in training even when they were ambivalent about cost, children's fatigue, or the narrowing of sport's educational meaning.

This distinction is important because the problem is not parental involvement itself. The findings suggest that parental involvement becomes problematic when children's sport participation is organized mainly around external returns, comparative anxiety, or purchasable advantages. In this situation, sport's educational value may be narrowed from enjoyment, bodily experience, cooperation, and sustained participation to test preparation, certificates, project status, and family display.

### 5.3 Inequality in youth sport opportunities: interpretive evidence and analytical boundary

The case further suggests that sport consumption can become a process through which family resources participate in the distribution of sport opportunities. High-quality training, specialist competitions, course renewal, private coaching, equipment, and good facilities often require economic, cultural, and informational resources. Within the limits of this study, differences in family resources appeared to shape differences in access to sustained sport support.

This argument is consistent with international research on socioeconomic disparities in organized youth sport. Studies have shown that family income, parental education, and class resources are associated with children's access to organized sport, club sport, and specialized sport pathways. The present study adds qualitative case evidence from urban China by showing how parents themselves interpreted unequal opportunities through fees, coaching quality, project hierarchy, competition participation, and the risk of falling behind.

At the same time, the analytical boundary of this claim should be made clear. This study did not systematically collect household income, parental education level, complete training-fee records, or school-provision indicators. Family capital was inferred qualitatively from occupations, narratives about affordability and training choices, observed consumption practices, project selection, training frequency, fee discussions, and access to coaching, venues, equipment, and competition opportunities. Therefore, the inequality argument should be understood as interpretive and exploratory rather than as a statistical measurement of socioeconomic inequality. Future studies should use multi-site designs and collect more systematic indicators of household background, training expenditure, school sport provision, and community sport access.

### 5.4 Case-based governance implications

The governance implications of this study should also be understood within the limits of the evidence. Because the empirical data came mainly from parent interviews and observation in one commercial youth sport training setting, the study does not directly evaluate schools, communities, regulators, or training providers. The following implications are therefore case-based suggestions generated from the findings and related policy literature, rather than comprehensive policy conclusions.

First, school physical education should remain a primary public site for youth sport participation. If school physical education and after-school sport services provide diverse, enjoyable, safe, and sustained opportunities for all students, families may feel less pressure to purchase additional training merely to compensate for insufficient public provision or to protect examination performance. Improving school sport provision may therefore help reduce overreliance on commercial training.

Second, community sport provision can offer lower-threshold opportunities outside both school and commercial clubs. Open sport venues, youth-friendly community sport spaces, public-interest sport courses, and accessible competitions may provide alternatives for families who cannot sustain high-cost training. Community provision is especially important when family resources shape access to coaching, venues, equipment, and competition opportunities.

Third, commercial youth sport training should be governed through clearer standards for coaching qualifications, curriculum quality, safety responsibilities, fee transparency, and advertising boundaries. The findings suggest that claims related to score improvement, course upgrading, competition opportunities, or guaranteed advantages may influence family decisions. Regulation should therefore pay attention not only to safety and service quality, but also to how training institutions communicate the value and expected outcomes of youth sport.

Finally, family sport education guidance is needed. Parents should be supported to understand sport's long-term value beyond scores, certificates, and displayable achievements. In choosing sport projects, families should listen to children's voices, attend to bodily experience and fatigue, and protect enjoyment and sustained participation. These implications point to a coordinated approach in which school provision, community access, market regulation, and family education work together to protect children's agency and participation equity.

### 5.5 Strengths and limitations

The study has several clearly defined strengths. First, it provides qualitative evidence from an everyday commercial youth sport training setting, a context in which family expectations, school-related sport assessment, peer comparison, and market provision intersect. Second, it combines participant observation, semi-structured interviews, and field notes, allowing the analysis to connect parents' narratives with observed interactions in the training environment. Third, by introducing jiwa-style parenting into youth sport consumption research, the study offers a culturally situated explanation of how sport may be incorporated into family educational planning. These strengths support the study's contribution to sport sociology, while remaining consistent with its case-study boundaries.

Several limitations should also be noted. First, the study is based on one market-oriented youth fitness club in Guangzhou and cannot be generalized directly to all regions, class groups, or sport-training contexts. Second, the data are mainly from parent perspectives; no children or adolescents were directly interviewed. Therefore, claims about children's fatigue, enjoyment, and agency are based on parental reports and observational impressions rather than children's own accounts. Third, the study did not systematically collect household income, parental education level, exact training-fee records, or school-provision indicators. Family capital and participation equity were therefore analyzed qualitatively rather than measured directly. Fourth, the study did not include interviews with school administrators, physical education teachers, community sport providers, regulators, or training-market managers. As a result, the governance discussion should be read as case-based implications rather than direct evaluation of these actors.

Future research can use multi-site designs, mixed methods, and comparisons across school, community, and commercial sport settings. It should also include children, adolescents, physical education teachers, coaches, training providers, and policy actors. Collecting more systematic indicators of household background, sport expenditure, school sport provision, and community sport access would help examine more precisely how family sport consumption relates to participation equity.

## 6 Conclusion

Based on 20 days of participant observation and interviews with 18 parents in one market-oriented youth fitness club in Guangzhou, this qualitative case study suggests that jiwa-style parenting appeared to reshape parents' understanding and organization of youth sport consumption within the observed commercial training setting. Sport was gradually interpreted by parents as family sport investment, symbolic distinction, risk management, and competition over opportunities.

Four interrelated processes were identified in this case. First, sport participation was converted into family investment as preparation for perceived future advantage. Second, sport projects, equipment, and competition experiences were symbolized as displays of family distinction and parenting competence. Third, sport training became pressure-driven under the combined influence of fitness tests, physical education examinations, peer comparison, and market promotion. Fourth, unequal family resources appeared to differentiate young people's access to coaching, venues, competitions, and sustained training support.

The study contributes to sport sociology by placing youth sport consumption within family education beliefs, family capital, and sport opportunity structures. It does not claim to represent youth sport consumption in urban China as a whole. Instead, it identifies a possible case-based mechanism through which family expectations, school-related assessment, peer comparison, and commercial training provision may interact. The significance of these findings lies in showing how youth sport consumption can become a site where family educational aspirations, commercial sport provision, and unequal access to sport opportunities intersect. Practically, this case-based insight may help readers understand why expanding youth sport participation requires attention not only to training opportunities, but also to children's agency, family burden, public sport provision, and equitable access.

## Data Availability

The dataset consists of qualitative interview transcripts and field notes that contain potentially identifiable private information about adult participants, their children, schools, the youth fitness club, and specific events. Public sharing is restricted to protect participant confidentiality and comply with the confidentiality assurances provided during informed consent. De-identified data may be made available from the corresponding author upon reasonable request, subject to privacy protection and ethical considerations. Requests to access the datasets should be directed to Yuanyuan Li liyuan018332@163.com.
